# Spatial and Temporal Association of Outbreaks of H5N1 Influenza Virus Infection in Wild Birds with the 0°C Isotherm

**DOI:** 10.1371/journal.ppat.1000854

**Published:** 2010-04-08

**Authors:** Leslie A. Reperant, Neven S. Fučkar, Albert D. M. E. Osterhaus, Andrew P. Dobson, Thijs Kuiken

**Affiliations:** 1 Department of Ecology and Evolutionary Biology, Princeton University, Princeton, New Jersey, United States of America; 2 Atmospheric and Oceanic Sciences Program, Princeton University, Princeton, New Jersey, United States of America; 3 Department of Virology, Erasmus Medical Centre, Rotterdam, the Netherlands; University of Oxford, United Kingdom

## Abstract

Wild bird movements and aggregations following spells of cold weather may have resulted in the spread of highly pathogenic avian influenza virus (HPAIV) H5N1 in Europe during the winter of 2005–2006. Waterbirds are constrained in winter to areas where bodies of water remain unfrozen in order to feed. On the one hand, waterbirds may choose to winter as close as possible to their breeding grounds in order to conserve energy for subsequent reproduction, and may be displaced by cold fronts. On the other hand, waterbirds may choose to winter in regions where adverse weather conditions are rare, and may be slowed by cold fronts upon their journey back to the breeding grounds, which typically starts before the end of winter. Waterbirds will thus tend to aggregate along cold fronts close to the 0°C isotherm during winter, creating conditions that favour HPAIV H5N1 transmission and spread. We determined that the occurrence of outbreaks of HPAIV H5N1 infection in waterbirds in Europe during the winter of 2005–2006 was associated with temperatures close to 0°C. The analysis suggests a significant spatial and temporal association of outbreaks caused by HPAIV H5N1 in wild birds with maximum surface air temperatures of 0°C–2°C on the day of the outbreaks and the two preceding days. At locations where waterbird census data have been collected since 1990, maximum mallard counts occurred when average and maximum surface air temperatures were 0°C and 3°C, respectively. Overall, the abundance of mallards (*Anas platyrhynchos*) and common pochards (*Aythya ferina*) was highest when surface air temperatures were lower than the mean temperatures of the region investigated. The analysis implies that waterbird movements associated with cold weather, and congregation of waterbirds along the 0°C isotherm likely contributed to the spread and geographical distribution of outbreaks of HPAIV H5N1 infection in wild birds in Europe during the winter of 2005–2006.

## Author Summary

Highly pathogenic avian influenza virus of the H5N1 subtype emerged more than a decade ago in poultry in South-East Asia. In 2005, it spread outside Asia infecting both poultry and wild birds in the Middle East, Europe and Africa. Both trade of poultry and movements of wild birds were likely implicated in the spread of the infection; however, the ability of wild birds to carry the virus to novel geographical areas is still highly debated and remains obscure. In Europe, the virus mainly infected wild birds, and emergence coincided with a spell of cold weather, which is known to result in massive movements of wild waterbirds. In this paper, we demonstrate that movements of wild waterbirds associated with cold weather contributed to the spread and geographical distribution of outbreaks in Europe during the winter of 2005–2006. Higher density of wild waterbirds on bodies of water that remain unfrozen ahead of the freezing line likely favoured transmission of the virus and resulted in distinctive distribution of outbreaks at locations where surface air temperatures were 0°C–2°C. This has important implications for surveillance, which should target areas where temperatures are close to freezing in winter, especially in poultry-dense regions close to areas where waterfowl aggregate.

## Introduction

Highly pathogenic avian influenza virus (HPAIV) H5N1 spread from Asia to Europe, the Middle East and Africa during the winter of 2005–2006, with sporadic outbreaks reported in poultry and wild bird populations. Both trade of poultry and poultry products and wild bird movements may have contributed to the unprecedented geographical spread of the virus [1],[2]. In Europe, most cases in wild bird populations occurred in areas where no outbreaks had previously been detected in poultry, indicating that wild birds were likely implicated in the spread of the infection [1]–[3]. Outbreaks of HPAIV H5N1 infection in Europe followed waterbird movements associated with a spell of cold weather [3]–[5], and the route of introduction of HPAIV H5N1 in western European countries was most likely associated with movements of wild birds [1],[2]. However, the role of wild birds in the spread of HPAIV H5N1 remains highly controversial [2],[6], and the association between movements of wild birds associated with cold weather and outbreaks of HPAIV H5N1 infection in Europe during the winter of 2005–2006 remains conjectural.

Experimental infections of naïve waterbirds with HPAIV H5N1 further supported a possible role of wild birds in the spread of the virus. Certain species are highly susceptible to developing clinical signs, may rapidly succumb to severe disease (e.g., swans, *Cygnus* spp., and European small diving ducks *Aythya* spp.), and will thus highlight local outbreaks without contributing significantly to long-distance transmission [7]–[9]. In contrast, other species show no visible signs, shed virus for several days, and potentially play a significant role as spreaders of HPAIV H5N1 (e.g., mallard, *Anas platyrhynchos*) [8]. Migratory and within-winter movements of such species asymptomatically infected with HPAIV H5N1 may result in significant geographical spread of the virus [8]. Outbreaks of HPAIV H5N1 infection in wild birds may then occur and only be detected when spreader species aggregate with highly susceptible species.

The migratory and wintering strategies of European waterbird species vary (as do those of sub-populations of some species) [10]. Waterbird species breeding at most northern latitudes are highly migratory, wintering at southern latitudes, including sub-Saharan Africa. These species include the Eurasian wigeon (*Anas Penelope*) and the northern pintail (*A. acuta*). In contrast, waterbird species breeding both at northern latitudes and in more temperate regions of Europe are partially migratory. While sub-populations breeding at most northern latitudes migrate to southern latitudes, sub-populations breeding at more temperate latitudes remain and winter in these regions. Such species include the common teal (*A. crecca*), the gadwall (*A. strepera*), the mallard, the common pochard (*Aythya ferina*) and the tufted duck (*A. fuligula*). The garganey (*Anas querquedula*) is one exception, as it is a fully migratory species, breeding in temperate regions of Europe and wintering exclusively in sub-Saharan Africa [10].

Waterbirds winter in areas where bodies of water remain unfrozen, allowing them to forage. Their wintering range is thus determined by the extent of ice cover [11]. For shallow fresh waters that dabbling ducks (*Anas* spp.) and lighter diving ducks (*Aythya* spp.) depend upon [11], the occurrence of ice cover is tightly coupled to surface air temperatures [12], and severe cold spells can drive massive within-winter movements of waterbirds [10],[13],[14]. Two wintering strategies are used by migratory waterbirds in response to cold weather. On the one hand, because cold spells can entail high energy expenditures and limit food availability, migratory waterbirds may winter at southern latitudes, where adverse weather conditions are rare [11]. On the other hand, because long migration distance from their wintering grounds to their breeding grounds impairs waterbirds' reproductive success [11], wintering waterbirds may minimize this distance by congregating on unfrozen bodies of water located as close as possible to their breeding grounds [15].

In recent years, sub-populations of migratory waterbird species that typically leave their breeding grounds in northern Europe for more southern wintering grounds have remained sedentary during relatively mild winters [10]. Furthermore, migratory waterbirds, such as the Eurasian wigeon and the common teal, winter in most southern locations, such as Spain and northwestern Africa, only during harsh winters [16]. Together with the large number of partially migratory waterbird species listed above, it suggests that the latter strategy is common in Europe. In addition, migratory waterbirds using the former strategy and wintering in more southern latitudes start their spring migration towards the breeding grounds as early as February [10],[17]. They may be slowed by cold weather, and constrained to stage on unfrozen bodies of water located at the forefront of the freezing front. All these conditions cause waterbirds to congregate during the winter period in suitable habitats along the freezing front where bodies of fresh water are not frozen. This will favour the transmission and spread of HPAIV H5N1 within and between species, resulting in outbreaks in wild bird populations [5]. Such transient aggregations of waterbirds close to populations of domestic poultry will increase the chance of pathogen spill-over into these hosts, which may eventually result in transmission into other domestic animals and humans.

We hypothesize that the initial cases of HPAIV H5N1 infection in wild birds in Europe during the winter of 2005–2006 occurred at locations where surface air temperatures were close to 0°C, because of higher waterbird densities along the freezing front. To test this hypothesis, we analysed the spatio-temporal correlation of surface air temperatures with the initial outbreaks of HPAIV H5N1 infection in wild birds in Europe during the winter of 2005–2006. The spatio-temporal correlation of surface air temperatures with waterbird abundance was also assessed, using publicly available data on maximum mid-January counts of mallards obtained between 1990 and 2003 across Europe (from the French Atlantic coast to eastern Ukraine and from Spain and Greece to Denmark), and mid-January counts of abundant duck species obtained between 1993 and 2008 in eastern France and Switzerland. Census sites in Eastern France and Switzerland were chosen because this region, often hit by cold spells and surface air temperatures close to 0°C, is highly used by wintering mallards and diving ducks [10]. Mallards and a proportion of common pochards experimentally infected with HPAIV H5N1 remained clinically healthy yet shed high viral titers, potentially spreading the virus over long distances [8]. Analyzing the relationship between the abundance of these species and surface air temperatures at these census sites may thus provide useful indications for higher waterbird densities close to the 0°C isotherm.

## Results

### Outbreaks of HPAIV H5N1 infection

A total of 52 locations in 15 European countries experienced initial outbreaks of HPAIV H5N1 infection in wild bird populations (Table S1). Initial outbreaks of HPAIV H5N1 infection in wild bird populations were defined as one or more cases of HPAIV H5N1 infection in wild birds in countries where the first reported case(s) occurred in wild birds, at locations with no outbreak in poultry or wild birds within ∼120 km in the preceding month. Only countries with first cases of HPAIV H5N1 infection in wild birds were selected to exclude cases resulting from introduction by and spill-back transmission from infected poultry nearby. Likewise, cases were selected at locations with no outbreak in poultry or wild birds within ∼120 km in the preceding month to exclude cases resulting from local spread independent of within-winter movement of wild birds. A perimeter of ∼120 km was used based on the typical range of dispersive movements of wild waterfowl of at least 100 km [10]. The date of initial outbreaks of HPAIV H5N1 infection in wild birds used in the analyses is the date infected birds were found moribund or dead.

**Figure 1:**
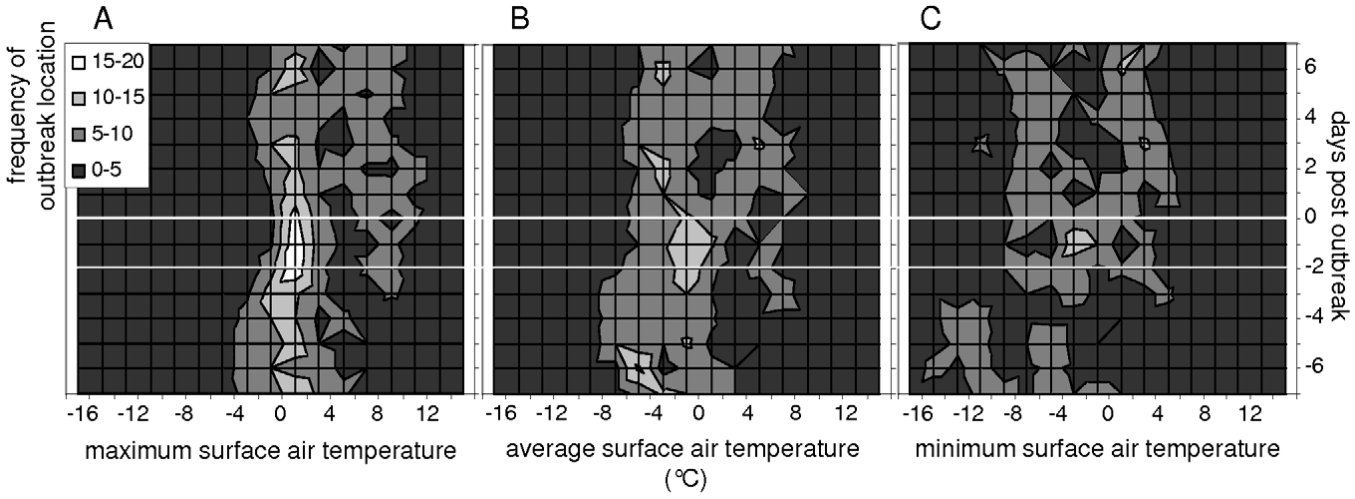
Spatio-temporal association of outbreaks of highly pathogenic avian influenza virus (HPAIV) H5N1 infection in wild birds with surface air temperatures. Frequency distributions of daily A) maximum, B) average, and C) minimum surface air temperatures (°C) at locations of initial outbreaks of HPAIV H5N1 infection in wild birds, from seven days before to seven days after wild birds infected with HPAIV H5N1 were found. Two white lines mark three distinct periods: from day −7 to day −3; from day −2 to day 0; and from day +1 to day +7. Day 0 corresponds to the date wild birds infected with HPAIV H5N1 were found. Outbreaks of HPAIV H5N1 infection in wild birds occurred most frequently when maximum surface air temperatures were around 2°C on day −2 to day 0 (A). A similar but less pronounced trend is observed for daily average and minimum surface air temperatures: average surface air temperatures at locations of initial outbreaks of HPAIV H5N1 infection in wild birds were most often around 0°C on day −2 to day 0 (B) and minimum surface air temperatures at these locations were most often around −2°C on day −2 to day 0 (C).

**Figure 2:**
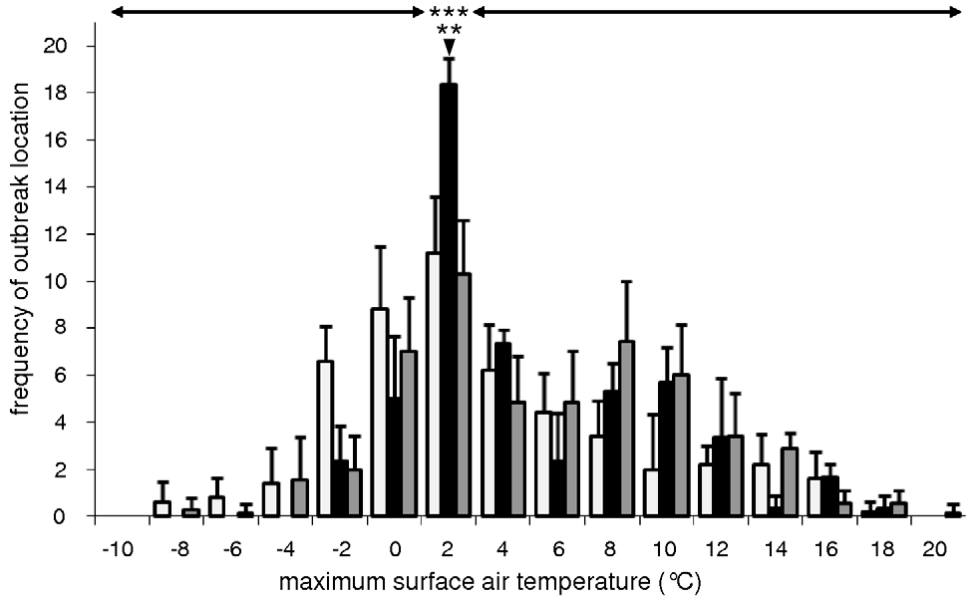
Significant association of outbreaks of highly pathogenic avian influenza virus (HPAIV) H5N1 infection in wild birds with maximum surface air temperatures of 0°C–2°C. Frequency distribution of daily maximum surface air temperatures (°C) at locations of initial outbreaks of HPAIV H5N1 infection in wild birds, from day −7 to day −3 (light grey), from day −2 to day 0 (black), and from day 1 to day 7 (dark grey). Standard bars represent standard deviations. Day 0 corresponds to the date wild birds infected with HPAIV H5N1 were found. Outbreaks of HPAIV H5N1 infection in wild birds occurred significantly more often at locations where daily maximum surface air temperatures were 0°C–2°C on day −2 to day 0 than at locations with daily maximum surface air temperatures of any other two-degree range during this period (ANOVA test, F = 9.5, p<0.0001; marked on the graph with *** and horizontal arrows). Daily maximum surface air temperatures of 0°C–2°C were more often recorded at the locations of initial outbreaks of HPAIV H5N1 infection in wild birds on day −2 to day 0 than at these locations on day −7 to day −3 and on day 1 to day 7 (ANOVA test, F = 15.1, p = 0.0005; marked on the graph with ** and arrow head).

Initial outbreaks of HPAIV H5N1 infection in wild bird populations occurred most often when surface air temperatures were close to 0°C (Fig. 1). Of the 52 locations, 17 to 19 (33%–37%) experienced maximum surface air temperatures of 0°C–2°C on the day wild birds infected with HPAIV H5N1 were found, and on the two preceding days (day −2 to day 0; Fig. 1A). In contrast, only 1 (2%) to 8 (15%) locations experienced maximum surface air temperatures within any other 2 degree-range between day −2 and day 0 (ANOVA test, F = 9.5, p<0.0001; Fig. 2). Furthermore, the number of locations experiencing maximum surface air temperatures of 0°C–2°C peaked to 17 to 19 on day −2 to day 0, while only 8 (15%) to 14 (27%) locations experienced maximum surface air temperatures of 0–2°C on day −7 to day −3 and day +1 to day +7 (ANOVA test, F = 15.1, p = 0.0005; Fig. 2). A similar but less pronounced trend was observed for minimum and average surface air temperatures (Fig. 1B and 1C).

Areas that did not experience surface air temperatures close to 0°C may have not reported HPAIV H5N1 outbreaks in wild birds due to reporting bias, for example associated with regional differences in the ornithological and bird-watching community, or national differences in resources allocated to surveillance programs. Because the intensity of surveillance of mortality events in wild birds may be linked to human population density in a region, we determined whether the reporting of initial outbreaks of HPAIV H5N1 infection in wild birds was biased towards more densely populated regions in Europe. The statistical distribution of population density of the first administrative regions that reported initial HPAIV H5N1 outbreaks in wild birds was not significantly different from that of regions across Europe (t = 0.58, p = 0.8; Fig. S1A). Because the intensity of surveillance of mortality events in wild birds may also be linked to a country's resources, we also determined whether the reporting of initial outbreaks of HPAIV H5N1 infection in wild birds was biased towards richer countries in Europe. Likewise, the statistical distribution of gross domestic product (GDP) per inhabitant of countries that reported initial HPAIV H5N1 outbreaks in wild birds was not statistical different from that in Europe (t = 0.25, p = 0.7; Fig. S1B).

### Duck abundance

**Figure 3:**
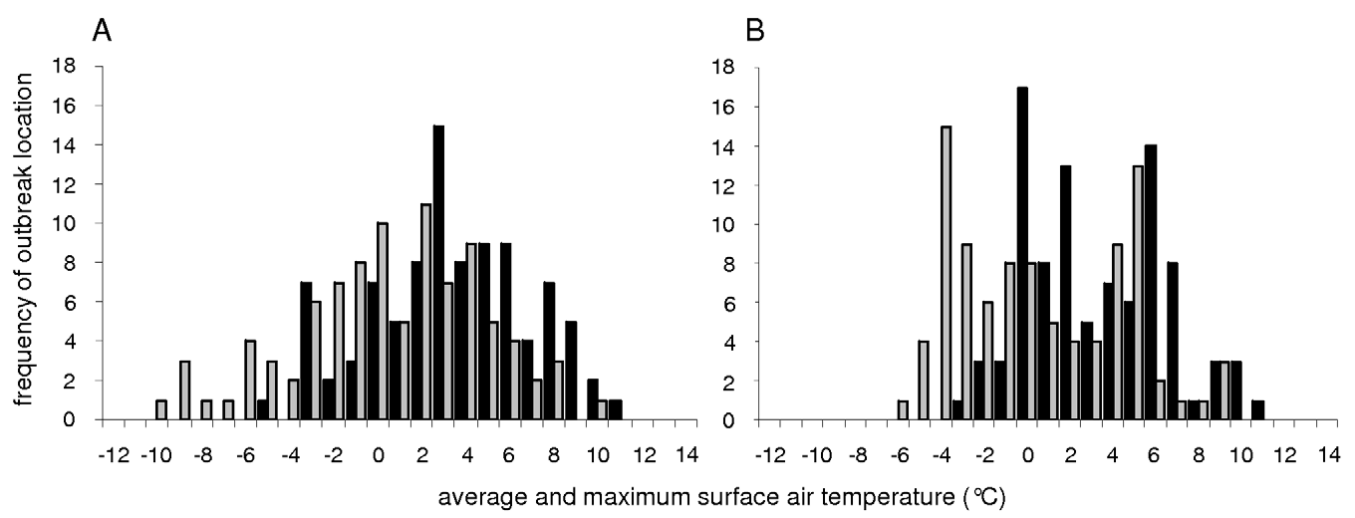
Surface air temperatures at locations of maximum mallard counts. Frequency distributions of average (grey) and maximum (black) mid-January surface air temperatures (°C) at each of 93 locations across Europe A) when maximum mid-January mallard counts were obtained (between 1990 and 2003), and B) over the period 1990–2003. Average and maximum mid-January surface air temperatures were normally distributed around a mean of 0°C and 3°C, respectively, at locations of maximum mid-January mallard counts, on the years maximum mid-January mallard counts were obtained. In contrast, average and maximum mid-January surface air temperatures were not normally distributed when averaged over the period 1990 and 2003 at each of these locations.

The highest counts of mallards obtained at each of 93 locations across Europe between 1990 and 2003 were typically recorded when average and maximum mid-January surface air temperatures were 0°C and 3°C, respectively. Average and maximum mid-January surface air temperatures (but not mid-January minimum surface air temperatures) at these locations on the years of maximum mallard counts were normally distributed, around a mean of 0.0°C and 2.9°C, with a standard deviation of 4.2°C and 3.6°C, respectively (Shapiro-Wilk normality test, W = 0.98 and W = 0.98; p = 0.2, and p = 0.3, respectively; Fig. 3A). In contrast, average and maximum mid-January surface air temperatures at these locations were not normally distributed when averaged over the entire period 1990–2003 for each location (Shapiro-Wilk normality test, W = 0.94 and W = 0.96; p = 0.0004 and p = 0.01, respectively; Fig. 3B).

Mallards, common pochards (*Aythya ferina*), and tufted ducks (*A. fuligula*) were the most abundant waterbird species counted at 25 locations in Rhône-Alpes (eastern France) between 1993 and 2008, and at 18 locations across Switzerland between 2002 and 2007. Average mid-January temperatures at these locations had a mean of 0.2°C (SD = 3.4°C). Minimum mid-January temperatures at these locations had a mean of −3.2°C (SD = 4.4°C). Maximum mid-January temperatures at these locations had a mean of 3.3°C (SD = 2.9°C).

**Figure 4:**
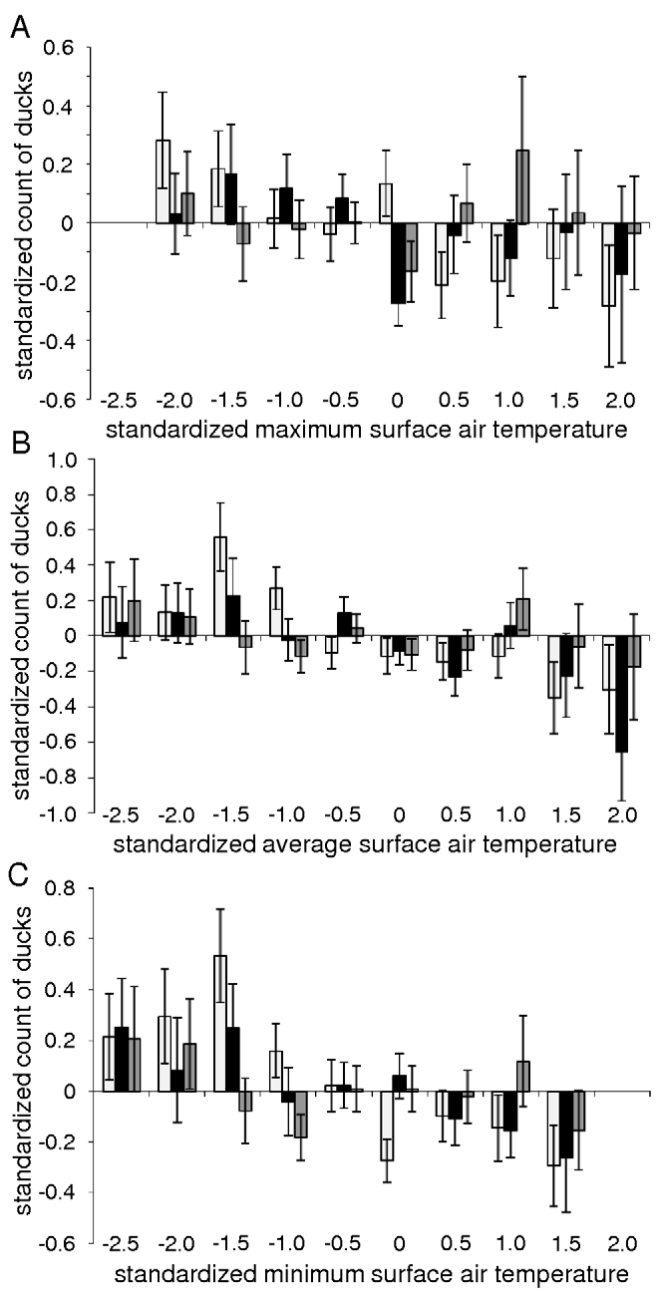
Relationship between duck abundance and surface air temperatures. Standardized mid-January counts of mallards (light grey), common pochards (black), and tufted ducks (dark grey) as a function of standardized A) maximum, B) average, and C) minimum mid-January surface air temperatures. Standard bars represent standard errors. Mallard and common pochard abundances were significantly negatively correlated with maximum, average, and minimum mid-January surface air temperatures based on linear and non-linear generalized least square models with an autocorrelation structure of first order (see Table 1).

Standardized mid-January counts of mallards and common pochards (but not tufted ducks) were negatively correlated with standardized minimum, average, and maximum mid-January surface air temperatures (Fig. 4), based on generalized least square linear and non-linear models with an autocorrelation structure of first order. These models determine the linear or non-linear equations of the form ax+b and ax^2^+bx+c, respectively, that best fit the data (and thus minimize the distance between points from the dataset and points generated by the models). An autocorrelation structure of first order was included because of the spatial correlation that exists between the data points (surface air temperatures at one location are not independent of those at another location within the region investigated). The goodness-of-fit of linear models were slightly higher than that of non-linear models (Table 1). This analysis provides evidence for an overall negative relationship between mallard and common pochard abundance and surface air temperatures. However, because of the lack of temporal precision (the exact mid-January count date was unknown and mid-January temperatures were averaged over a period of 10 days around January 15^th^), more detailed conclusion on the relationship between duck abundance and ice cover likelihood at the time of the count cannot be drawn. Interestingly, higher counts of mallards and common pochards were typically recorded when surface air temperatures were slightly below the mean temperatures of the region, and their abundance tended to decrease as surface air temperatures further decreased (Fig. 4).

**Table 1:** Correlation between mid-January waterbird counts and mid-January surface air temperatures determined by linear and non-linear generalized least square models with an autocorrelation structure of first order.

			Maximum surface air temperature	Average surface air temperature	Minimum surface air temperature
Mallard	Linear model	Coefficient a	−0,12	−0,17	−0,18
		Coefficient b	−0.01	−0.02	−0.02
		df	4	4	4
		BIC	1231	1224	1221
		AIC	1214	1208	1205
		*p*	***0,007***	***0,0002***	***0,0001***
	Non-linear model	Coefficient a	0,01	−0,01	−0,02
		Coefficient b	−0,12	−0,17	−0,19
		Coefficient c	−0,02	−0,01	0.00
		df	5	5	5
		BIC	1237	1230	1227
		AIC	1216	1210	1207
		*p*	***0,007***	***0,0004***	***0,0001***
Common pochard	Linear model	Coefficient a	−0,09	−0,10	−0,10
		Coefficient b	−0.01	−0.01	−0.01
		df	4	4	4
		BIC	1181	1180	1180
		AIC	1165	1164	1164
		*p*	***0,04***	***0,03***	***0,03***
	Non-linear model	Coefficient a	0,01	−0,01	−0,03
		Coefficient b	−0,10	−0,11	−0,12
		Coefficient c	−0,02	0,01	0,02
		df	5	5	5
		BIC	1187	1186	1186
		AIC	1166	1166	1166
		*p*	***0,04***	***0,03***	***0,03***
Tufted duck	Linear model	Coefficient a	0,01	−0,01	−0,02
		Coefficient b	0.00	0,00	0.00
		df	4	4	4
		BIC	1145	1145	1145
		AIC	1129	1129	1129
		*p*	*0,8*	*0,7*	*0,6*
	Non-linear model	Coefficient a	0,04	0,04	0,03
		Coefficient b	0,005	0,003	0,0004
		Coefficient c	−0,04	−0,04	−0,03
		df	5	5	5
		BIC	1150	1150	1150
		AIC	1130	1130	1130
		*p*	*0,9*	*0,9*	*0,9*

Significant p-values are shown in bold.

df is the degree of freedom; BIC is the Bayesian information criterion and AIC is the Akaike information criterion, which measure the goodness-of-fit of the models and are used for the selection of the best models (models with lowest BIC and AIC).

## Discussion

In the present paper, we show an association between the timing and locations of initial outbreaks of HPAIV H5N1 infection in wild birds in Europe during the winter of 2005–2006 and the 0°C isotherm. Initial outbreaks of HPAIV H5N1 infection in wild birds occurred significantly more often at locations where maximum surface air temperatures were between 0°C and 2°C, on the day of the outbreaks and the two preceding days. When visualized on a dynamical map showing daily movements of maximum surface air temperature isotherms between January and March 2006 in Europe, most initial outbreaks of HPAIV H5N1 in wild birds occur within an area delineated by the 0°C and 2°C isotherms, and appear to closely follow the surge and ebb of the cold front (Video S1). Because surveillance of avian influenza in wild birds has been up-scaled in Member States of the European Union since 2002, and in most of Europe following the introduction of HPAIV H5N1 in 2005 [18], it is unlikely that many outbreaks went unnoticed outside the geographical range of the 0°C isotherm during the winter of 2005–2006. Furthermore, the absence of significant difference between human population density and GDP per inhabitant in regions or countries where outbreaks were reported and those across Europe implies that surveillance efforts were not significantly biased towards more densely populated regions or richer countries. Therefore, the geographical distribution of reported HPAIV H5N1 outbreaks in wild birds in Europe along the 0°C isotherm is likely representative of a real and important phenomenon that should be used to refocus surveillance for future outbreaks.

Waterbirds congregate in winter on unfrozen bodies of fresh water where they can forage [10],[11], and higher waterbird densities may occur along the freezing front. On the one hand, waterbirds may choose to keep as close as possible to their breeding grounds to maximize their reproductive success, and may be displaced by cold fronts; on the other hand, waterbirds may choose to migrate to and winter in regions with rare adverse weather conditions, and may be slowed by cold fronts upon their journey back to the breeding grounds, which can start as early as February [10],[11],[17]. Maximum counts of mallards across continental Europe typically occurred when average and maximum surface air temperatures were 0°C and 3°C at the census location, respectively. This suggests that temperatures slightly above 0°C are one factor favouring maximum numbers of mallards at wintering locations, notably in central and northern Europe, where such winter temperatures are frequently recorded. Overall, mallard and common pochard counts at 43 locations across eastern France and Switzerland were negatively correlated with surface air temperatures. Higher numbers of mallards and common pochards occurred when mid-January surface air temperatures were lower than mean mid-January temperatures of the region. These results strongly imply the existence of a relationship between duck abundance and surface air temperatures, despite the coarse quality of mid-January counts and mid-January surface air temperatures. Nevertheless, a number of confounding factors, such as the abundance of food resources, waterbird breeding success in the preceding spring and summer, or changes in wetland management and disturbance, likely impact on yearly duck counts in Europe and could not be accounted for due to the nature of the data. Detailed studies of the correlation of long-term daily counts of waterbirds in multiple sites across Europe in winter with daily surface air temperatures are needed to further assess whether the abundance of wintering ducks is higher when surface air temperatures are close to 0°C. Although limited data are available on the association between waterbird abundance and surface air temperature, it is interesting to note that, for instance, lower mean temperatures in the Dombes region in France in the winter of 2005–2006 were associated with unusual early arrival of common pochards [4]. Likewise, lasting cold weather was associated with higher waterbird densities in eastern and southern Germany [5]. On the other hand however, the abundance of tufted ducks was not correlated with surface air temperatures. Ice cover impacts on the distribution and abundance of dabbling and lighter diving ducks in winter, because they require shallow waters to forage [11]. While common pochards feed on both animal prey and plants, tufted ducks feed predominantly on molluscs and thus forage typically in deeper waters than common pochards [19], possibly explaining their lower sensitivity to surface air temperatures, as found in this study.

Maximum surface air temperature close to 0°C, yet on the positive side of the isotherm, may provide wintering conditions leading to maximum congregation of waterbirds, notably Anatidae, and so favour influenza virus transmission. Cold fronts and freezing temperatures are typically associated with anticyclonic conditions and (north-)easterly winds in Europe [20]. Maximum surface air temperatures above 0°C, despite minimum surface air temperatures below 0°C, thus may determine the absence of ice cover on inland bodies of fresh water located (south-)westward from the cold front. Such temperature conditions were prevalent at locations with initial outbreaks of HPAIV H5N1 infection in wild birds in Europe during the winter of 2005–2006. Therefore, congregation of waterbirds asymptomatically infected with HPAIV H5N1 and highly susceptible waterbird species in suitable habitats along the freezing front likely promoted the onset of outbreaks during the winter of 2005–2006 in Europe. Accordingly, densities of mute swans (*Cygnus olor*) and Anatidae were three to six times higher in ponds of the Dombes region where outbreaks of HPAIV H5N1 infection occurred, than in unaffected ponds [4]. Likewise, in eastern and southern Germany, where outbreaks of HPAIV H5N1 infection occurred, high waterbird densities were recorded on bodies of fresh water that remained un- or partially frozen. In contrast, waterbird densities were lower in western Germany, which experienced milder weather conditions, and no cases of HPAIV H5N1 infection in wild birds [5].

Higher densities of waterbirds along the freezing front likely favoured increased transmission of HPAIV H5N1. Excretion of HPAIV H5N1 in experimentally infected waterbirds starts as soon as one day following inoculation and peaks between 1 and 3 days following inoculation, typically lasting 5 days [7]–[9]. While the dabbling ducks tested did not develop clinical disease at all, diving ducks, as well as mute and whooper swans, can develop clinical disease as soon as 2 days, and die as soon as 4 days following inoculation [7],[8], although longer incubation period and longer time to death have been described in swans [9]. Clinical disease and death may occur early during the course of infection in free-ranging waterbirds due to contributing factors, such as poor nutritional and health status or harsh environmental conditions. Thus, there may be a short time-lag of 2 to 4 days between transmission of HPAIV H5N1 to uninfected birds, and death and report of HPAIV H5N1 outbreaks in wild waterbirds. Maximum surface air temperatures of 0°C–2°C were most frequently reported at locations of HPAIV H5N1 outbreaks up to two days before the day birds were found dead, which likely accounted for the time-lag between aggregation of wild waterbirds, transmission of HPAIV H5N1 and report of morbidity or mortality. Furthermore, because the peak of the infectious period is typically short, aggregation of waterbirds during two days along the 0°C isotherm may be sufficient to result in sustained HPAIV H5N1 transmission and detectable outbreaks.

Alternatively, and potentially concomitantly, maximum surface air temperatures close to 0°C may favour the persistence of HPAIV H5N1 in the environment and enhance environmental transmission of the virus independently of waterbird density. Avian influenza viruses (AIV) have been experimentally shown to remain infective for several months in water at low temperatures (below 17°C) and low salinity levels (fresh- or brackish water) [21],[22]. Environmental transmission of LPAIV is increasingly recognized as essential in maintaining LPAIV locally and from year to year [23]–[25]. However, little is known on the role environmental transmission had on HPAIV H5N1 dynamics in Europe, and several arguments in support of environmental transmission of HPAIV H5N1 can be raised. First, HPAIV H5N1 belonging to the lineage isolated in wild waterbirds in Europe remained infective for 158 days in fresh water at 17°C, and for 26 days at 28°C [26]. Second, although HPAIV H5N1 are mainly excreted from the respiratory tract of infected waterbirds, potentially favouring direct density-dependent transmission [8], this does not preclude contamination of lake water by respiratory excretions or infected carcasses, allowing environmental transmission of the virus. Third, assuming persistence patterns similar to those of LPAIV [21], slower loss of infectivity at lower temperatures may have contributed to the geographical distribution of HPAIV H5N1 outbreaks along the freezing front. However, persistence of more than 4 months in fresh water at 17°C strongly suggests that environmental transmission of HPAIV H5N1 would occur even in warmer water away from the 0°C isotherm. Furthermore, freezing and thawing are known to substantially decrease AIV infectivity by up to 10 fold, despite having little effect on AIV RNA [27]. Thus, LPAIV RNA has been recovered in ice from Siberian lakes, yet no infectious virus could be isolated [28]. Therefore, although environmental transmission cannot be ruled out, it would most likely result in a more uniform distribution of HPAIV H5N1 outbreaks where bodies of water remained unfrozen, and thus is unlikely to account alone for the geographical distribution of HPAIV H5N1 outbreaks at locations where maximum surface air temperatures were close to 0°C.

Certain waterbird species do not develop clinical disease upon experimental infection with HPAIV H5N1, and may play a crucial role as reservoirs or spreaders of infection [8]. Conversely, asymptomatic infection of wild waterbirds with low pathogenic avian influenza viruses is speculated to result in costs that may hinder the ability of infected birds to fly over long distances [29],[30]. Also, because of the relatively short infectious period of avian influenza virus infection in waterbirds, infected waterbirds may not disperse avian influenza viruses over long distances [30]. Therefore, the role of migratory birds in the long-distance spread of HPAIV H5N1 outside Asia is still highly debated [2],[6]. However, distance, duration and efforts associated with within-winter movements of wild waterbirds are smaller than that associated with spring and autumn migratory flights, and within-winter movements can frequently be undertaken by waterbirds [10],[13],[14]. Although the poultry trade may have introduced HPAIV H5N1 into Russia, the Middle East or eastern Europe in fall and early winter of 2005–2006 [1],[3], within-winter waterbird movements associated with movements of the 0°C isotherm, following the surge and ebb of a cold spell that originated in the Black Sea area [3],[5], were likely undertaken by waterbirds asymptomatically infected with HPAIV H5N1. In conclusion, waterbird movements associated with cold weather and congregation of waterbirds along the 0°C isotherm likely contributed to the spread and geographical distribution of outbreaks of HPAIV H5N1 infection in wild birds in Europe during the winter of 2005–2006.

Movements of cold weather fronts, and in particular the 0°C isotherm of maximum surface air temperature, can readily be anticipated by operational weather forecasts. Therefore, increased active surveillance of HPAIV H5N1 infection in waterbirds should target populations occurring in areas where maximum surface air temperatures are close to freezing temperatures, i.e., along the positive side of the 0°C isotherm. Such targeted surveillance should pay special attention to poultry-dense areas, as this may increase the chance of detecting early the presence of HPAIV H5N1 in both wild and domestic bird populations in Europe during winter.

## Materials and Methods

### Data

#### Outbreaks of HPAIV H5N1 infection

The locations and dates of initial cases of HPAIV H5N1 infection in wild birds that occurred in Europe during the winter of 2005–2006 (between 19 October 2005 and 31 March 2006) were obtained from the World Organization for Animal Health (OIE; [31]).

#### Duck abundance

Maximum mid-January counts of mallards recorded around January 15^th^ at each of 93 locations across Europe between 1990 and 2003 were obtained from Wetlands International [32]. Mid-January waterbird counts performed around January 15^th^ and coordinated by Wetlands International [33] were obtained from Rhône-Alpes Ornithological Center for 25 locations across the Rhône-Alpes region (eastern France) from 1993 to 2008, and from Sempach Ornithological Station for 18 locations across Switzerland from 2002 to 2007 (Table S2).

#### Surface air temperatures

Daily minimum, average, and maximum surface air temperatures recorded 2 metres above ground were obtained from the NCEP-NOE Reanalysis 2 [34]. Daily surface air temperatures at the locations of initial outbreaks of HPAIV H5N1 infection in wild birds were obtained from seven days before to seven days after the day wild birds infected with HPAIV H5N1 were found. Mid-January surface air temperatures at the locations of duck census were averaged over the period January 10^th^ to January 20^th^, for each year of duck census data, as well as for all years between 1990 and 2003 at the locations of maximum mallard counts.

#### Human population density and GDP per inhabitant

Human population density in first administrative divisions of European countries in 2006 was obtained from European Commission's database Eurostat (http://epp.eurostat.ec.europa.eu). European countries' GDP per inhabitant were obtained from International Monetary Fund's world economic outlook database (http://www.imf.org/external/pubs/ft/weo/2009/02/weodata/index.aspx).

### Analysis

All statistical analyses were performed in the R language [35]. Statistical differences are considered significant when p<0.5.

#### Outbreaks of HPAIV H5N1 infection

A total of 52 locations in 15 European countries experienced initial outbreaks of HPAIV H5N1 infection in wild bird populations, and were included in the analysis (Table S1). The spatio-temporal correlation of initial outbreaks of HPAIV H5N1 infection in wild birds with daily minimum, average, and maximum surface air temperatures at these locations was investigated using three-dimensional maps. ANOVA test was used to determine the significance of observed spatial and temporal differences in surface air temperatures at locations of initial outbreaks of HPAIV H5N1 infection.

#### Duck abundance

The frequency distribution of minimum, average and maximum surface air temperatures at each of 93 locations across Europe where maximum counts of mallards were obtained between 1990 and 2003 was analysed. It was compared with the frequency distribution of minimum, average and maximum surface air temperatures at each of these locations averaged over the entire period 1990 to 2003. Mid-January counts of mallards, common pochards, and tufted ducks obtained at 43 locations across Rhône-Alpes and Switzerland between 1993 and 2008 were standardized at each location, by subtracting the mean count to the actual count, and dividing it by the standard deviation (both mean and standard deviation were calculated at each location). This allowed for comparison of the variations in number of ducks across years and locations. Mallards, common pochards and tufted ducks were chosen because they were the most abundant dabbling and lighter diving ducks in Rhône-Alpes and Switzerland. Daily minimum, average, and maximum surface air temperatures were standardized over the entire period and region. In order to determine whether duck abundance was correlated with surface air temperatures, linear and non-linear regression analyses were performed using generalized least square models with an autocorrelation structure of first order.

#### Human population density and GDP per inhabitant

The statistical distributions of regional human population density and of national GDP per inhabitant in countries that reported initial outbreaks of HPAIV H5N1 in wild birds were compared with those of all European countries to determine whether there were statistically significant differences, using the Student t-test.

## Supporting Information

Figure S1 Comparison of the statistical distributions of regional human population density (A) and national gross domestic product per inhabitant (B) across Europe and in regions or countries that reported outbreaks of highly pathogenic avian influenza virus (HPAIV) H5N1 infection in wild birds. Box plots represent the first and third quartiles (box) and minimum and maximum values (whiskers). Horizontal axis is on a log scale and measures inhabitants per square kilometre (A) or United States dollars (B). No statistically significant difference was found.
(0.01 MB PDF)

Table S1 Locations and dates of initial outbreaks of highly pathogenic avian influenza virus (HPAIV) H5N1 infection in wild birds in Europe during the winter of 2005–2006 [31]; in chronological order.
(0.01 MB PDF)

Table S2 Locations of maximum mallard counts obtained at each of 93 locations across Europe between 1990 and 2003 [32], and locations of mid-January waterbird counts obtained in Rhône-Alpes (eastern France) between 1993 and 2008, and in Switzerland between 2002 and 2007 (available online at http://coraregion.free.fr and www.vogelwarte.ch, respectively). IWC: International Waterbird Census.
(0.02 MB PDF)

Video S1 Movie of the movements of maximum surface air temperature isotherms in Europe from January 26th to March 31st 2006 with locations of initial outbreaks of highly pathogenic avian influenza virus (HPAIV) H5N1 infection in wild birds indicated as small black spots. Large black spots mark the locations of such outbreaks on day 2 to 1 before wild birds infected with HPAIV H5N1 were found. Large white spots mark the locations of such outbreaks on the day wild birds infected with HPAIV H5N1 were found.
(9.45 MB WMV)
