# Supplementary material for: Spatial and Temporal Association of Outbreaks of H5N1 Influenza Virus Infection in Wild Birds with the 0°C Isotherm
Source: PLoS Pathog. 2010 Apr 8;6(4):e1000854. doi: 10.1371/journal.ppat.1000854 (PMC2851735; doi:10.1371/journal.ppat.1000854)
Supplement: Figure S1 — Comparison of the statistical distributions of regional human population density (A) and national gross domestic product per inhabitant (B) across Europe and in regions or countries that reported outbreaks of highly pathogenic avian influenza virus (HPAIV) H5N1 infection in wild birds. Box plots represent the first and third quartiles (box) and minimum and maximum values (whiskers). Horizontal axis is on a log scale and measures inhabitants per square kilometre (A) or United States dollars (B). No statistically significant difference was found. (0.01 MB PDF) [file ppat.1000854.s001.pdf]

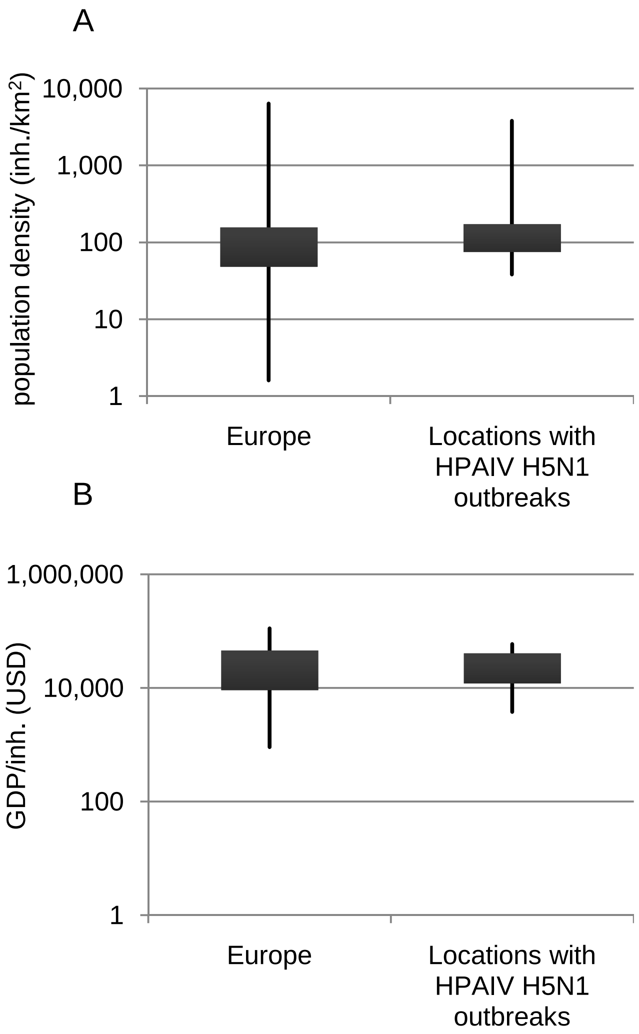

Supplementary Figure 1

Comparison of the statistical distributions of regional human population density (A) and national gross domestic product per inhabitant (B) across Europe and in regions or countries that reported outbreaks of highly pathogenic avian influenza virus (HPAIV) H5N1 infection in wild birds. Box plots represent the first and third quartiles (box) and minimum and maximum values (whiskers). Horizontal axis is on a log scale and measures inhabitants (inh.) per square kilometre (A) or United States dollars (B). No statistically significant difference was found.
