# Supplementary material for: Spatial and Temporal Association of Outbreaks of H5N1 Influenza Virus Infection in Wild Birds with the 0°C Isotherm
Source: PLoS Pathog. 2010 Apr 8;6(4):e1000854. doi: 10.1371/journal.ppat.1000854 (PMC2851735; doi:10.1371/journal.ppat.1000854)
Supplement: Table S1 — Locations and dates of initial outbreaks of highly pathogenic avian influenza virus (HPAIV) H5N1 infection in wild birds in Europe during the winter of 2005–2006 [31]; in chronological order. (0.01 MB PDF) [file ppat.1000854.s002.pdf]

Table S1

Locations and dates of initial outbreaks of HPAIV H5N1 infection in wild birds in Europe during the winter of 2005-2006 [31]; in chronological order.

Country	Latitude	Longitude	Date
Croatia	45.57	17.94	19-oct-05
Croatia	45.65	18.18	19-nov-05
Greece	40.27	22.58	30-jan-06
Bulgaria	43.76	22.71	31-jan-06
Italy	37.45	14.15	1-feb-06
Greece	38.93	24.55	3-feb-06
Greece	40.63	27.95	3-feb-06
Greece	40.97	25.15	6-feb-06
Italy	38.88	16.42	6-feb-06
Germany	54.63	13.23	8-feb-06
Slovenia	46.36	15.40	9-feb-06
Italy	40.87	16.60	14-feb-06
Bosnia and Herzegovina	44.35	17.19	16-feb-06
Slovakia	48.10	17.34	17-feb-06
Italy	42.83	12.53	19-feb-06
Germany	47.67	11.50	20-feb-06
Germany	48.88	12.59	20-feb-06
Germany	49.64	10.90	20-feb-06
Germany	52.95	9.91	20-feb-06
Croatia	43.51	16.24	21-feb-06
France	45.98	4.98	26-feb-06
France	43.45	5.01	28-feb-06
France	46.26	6.08	28-feb-06
Poland	52.24	15.47	1-mar-06
Poland	53.64	16.16	2-mar-06
Croatia	45.83	18.79	3-mar-06
Serbia and Montenegro	44.12	19.51	4-mar-06
Germany	47.91	12.11	5-mar-06
Germany	48.03	10.86	5-mar-06
Switzerland	47.69	8.64	6-mar-06
Slovenia	46.57	15.62	10-mar-06
Denmark	55.12	11.81	12-mar-06
Germany	53.22	13.81	14-mar-06
Germany	53.96	9.51	17-mar-06
Germany	54.02	12.08	17-mar-06
Germany	54.35	13.37	17-mar-06
Germany	47.59	9.68	18-mar-06
Czech Republic	49.05	14.43	20-mar-06
Denmark	55.97	9.77	24-mar-06
Germany	49.34	12.23	24-mar-06
Germany	49.43	11.06	24-mar-06
Denmark	54.86	9.99	25-mar-06
Denmark	55.25	14.74	26-mar-06
Denmark	55.25	11.27	27-mar-06
Germany	48.88	12.59	27-mar-06

Germany	53.77	11.27	27-mar-06
Poland	53.26	18.26	27-mar-06
UK	56.22	-2.73	30-mar-06
Germany	49.49	8.51	31-mar-06
Germany	49.54	10.43	31-mar-06
Germany	52.53	13.36	31-mar-06
Germany	54.22	9.77	31-mar-06
