# Supplementary material for: Spatial and Temporal Association of Outbreaks of H5N1 Influenza Virus Infection in Wild Birds with the 0°C Isotherm
Source: PLoS Pathog. 2010 Apr 8;6(4):e1000854. doi: 10.1371/journal.ppat.1000854 (PMC2851735; doi:10.1371/journal.ppat.1000854)
Supplement: Table S2 — Locations of maximum mallard counts obtained at each of 93 locations across Europe between 1990 and 2003 [32], and locations of mid-January waterbird counts obtained in Rhône-Alpes (eastern France) between 1993 and 2008, and in Switzerland between 2002 and 2007 (available online at http://coraregion.free.fr and www.vogelwarte.ch, respectively). IWC: International Waterbird Census. (0.02 MB PDF) [file ppat.1000854.s003.pdf]

Table S2

Locations of maximum mallard counts obtained at each of 93 locations across Europe between 1990 and 2003 [32], and locations of mid-January waterbird counts obtained in Rhône-Alpes (eastern France) between 1993 and 2008, and in Switzerland between 2002 and 2007 (available online at http://coraregion.free.fr and www.vogelwarte.ch, respectively). IWC: International Waterbird Census

Country	IWC site	Latitude	Longitude	Year
Croatia	Dunav: Batina	45.87	18.83	1990
Germ./Switz./Austr.	Bodensee Gesamt	47.59	9.47	1990
Germany	Rhein: Breisach - Nonnerweir	48.03	7.58	1990
Germany	Rhein: Nonnenweier-Kehl	48.35	7.76	1990
Germany	Stausee Dachwig	51.08	10.86	1990
Germany	Talsperre Spremberg	51.65	14.41	1990
Hungary	Bugyi	47.23	19.15	1990
Romania	Lake Sinoe	44.58	28.83	1990
Spain	Laguna de Gallocanta (Z)	41.00	-1.51	1990
Ukraine	Black Sea	46.48	30.76	1990
Ukraine	Cherkassi (Region)	49.41	32.06	1990
Ukraine	Crimea (Region)	44.93	34.10	1990
Ukraine	Poltava (Region)	49.59	34.55	1990
Denmark	Danish Wadden Sea	55.47	8.45	1991
Denmark	Lolland NW	54.73	11.46	1991
Germany	Weser: Strohause Plate	53.67	8.39	1991
Romania	Golesti	45.66	27.16	1991
Ukraine	Sasyk Liman	45.18	33.50	1991
Croatia	Ribnjacarstvo (Slavonski Brod Fishpond)	45.16	18.02	1992
Germany	Elbe Km	51.15	13.71	1992
Germany	Rhein: Weil-Breisach	50.31	8.42	1992
Hungary	Duna 02:00 Baja-Dunafoldvar	46.18	18.95	1992
Italy	Laguna D Di Grado E marano	45.71	13.35	1992
Spain	Tablas de Daimiel (Cr)	39.12	-3.74	1992
Ukraine	Vinitsa (Region)	49.23	28.49	1992
Bulgaria	Garvanovo Dam	41.97	25.45	1993
Germany	Wieck-Kooser See	54.15	13.40	1993
Hungary	Soponyai-Halastavak	47.01	18.46	1993
Netherlands	Eemmeer. Nijkerkernauw en Nuldernauw	52.25	5.43	1993
Poland	Odra: Krzepkowice-Brzeg Dolny	50.47	17.97	1993
Ukraine	Donetsk (Region)	48.03	37.75	1993
Ukraine	Zaporozhye (Region)	47.84	35.17	1993
Czech Republic	Nove Mlyny I & lidam Systems	48.86	16.73	1994
Hungary	Csaj. Pusztaszer (Halasto)=Tomorkeny	46.61	20.04	1994
Hungary	Hortobagy Halasto	47.58	21.15	1994

Romania	Delta Dunarii (Danube)	45.19	29.27	1994
Slovakia	Zemplinska Sirava reservoir	48.79	22.01	1994
Spain	Aiguamolls de l'emporda (Gi)	42.22	3.11	1994
Ukraine	Dnepropetrovsk (Region)	48.42	35.14	1994
Ukraine	Kharkov (Region)	49.99	36.26	1994
Ukraine	Kherson (Region)	46.70	32.66	1994
Czech Republic	Nechranice Dam	50.37	13.42	1995
Germany	Vorland Jadebusen	53.48	8.22	1995
Greece	Karla Reservoirs	39.44	22.79	1995
Greece	Kerkini	41.22	23.09	1995
Romania	Strejesti	44.53	24.27	1995
Hungary	Balaton: Total	48.10	20.32	1996
Hungary	Sumony Halasto	45.97	17.92	1996
Poland	Zbiornik Dzierzno	53.92	19.35	1996
Serbia	Jazovo Fish Pond	45.89	20.23	1996
Ukraine	Lake Kitai	45.35	33.16	1996
Bulgaria	Shabla Lake (+ Ezeretz)	43.57	28.57	1997
Yugoslavia	Uzdin-Idvor-Sakule	45.20	20.63	1997
Croatia	Kopacki Rit	45.63	18.89	1998
Germany	Dümmer	53.57	11.21	1998
Bulgaria	Danube: Russe-Tutrakan	43.85	25.95	1999
Bulgaria	Danube: Tutrakan-Silistra	44.12	27.26	1999
Croatia	Park Prirode Lonjsko Polje	45.42	16.65	1999
Greece	Axios, Loudias & Aliakmon Deltas	40.68	22.54	1999
Hungary	Biharugrai Halasto	46.97	21.60	1999
Netherlands	Grevelingen	51.76	3.92	1999
Netherlands	Groningse Noordkust	53.49	6.56	1999
Netherlands	Hollands Diep	51.70	4.51	1999
Netherlands	Ijssel	52.27	6.03	1999
Netherlands	Ijsselmeer	52.80	5.32	1999
Netherlands	Tjeukemeer	52.96	5.83	1999
Slovenia	Drava: Ptuj	46.42	15.87	1999
Ukraine	Area Northeast of Odessa	46.48	30.73	1999
Belarus	Minsk Town Waterbodies	53.97	27.58	2000
Bulgaria	Durankulak Lake	43.66	28.55	2000
France	Dombes-Vallée de l'Ain	45.97	5.00	2000
Greece	Evros Delta	41.24	26.14	2000
Hungary	Duna 01:00 Orsz. Hatar (=Border)-Baja	47.68	17.64	2000
Ukraine	Secondary delta of the Kiliya Channel (Danube)	45.45	29.26	2000
Austria	March	48.43	16.83	2001
Netherlands	Dollard	53.29	7.13	2001
Netherlands	Haringvliet	51.79	4.19	2001
Netherlands	Krimpenerwaard	51.98	4.78	2001
Netherlands	Markermeer	52.54	5.27	2001
Netherlands	Oosterschelde	51.64	3.84	2001
Netherlands	Texel	53.10	4.81	2001
Netherlands	Veerse Meer	51.53	3.69	2001
Netherlands	Westerschelde	51.38	3.87	2001
France	Baie de l'Aiguillon	46.28	-1.22	2002

France	La Camargue	43.52	4.60	2002
Germany	Donau: Km	48.60	10.86	2002
Hungary	Geszt. Begecsi Halastavak	47.44	21.32	2002
Hungary	Nagyhegyes Elepi-H.To	47.54	21.35	2002
Hungary	Retszilas Halasto	46.83	18.64	2002
Italy	Laguna Di Venezia	45.34	12.28	2002
France	Etangs de la Brenne	46.75	1.22	2003
France	Lac de Grandlieu	47.10	-1.68	2003
Spain	Parque Natural de la Alburfera	39.30	-0.32	2003
France	Beauvoir	45.12	5.34	1993-2008
France	Château-neuf Rochemaure	44.59	4.71	1993-2008
France	Cordon Rhône: Villebois	45.85	5.43	1993-2008
France	Donzère Mondragon	44.44	4.71	1993-2008
France	Etangs de la Dombes	45.97	5.00	1993-2008
France	Etangs de la plaine du Forez	45.70	4.18	1993-2008
France	Grangent	45.46	4.26	1993-2008
France	La Vanelle	45.03	4.98	1993-2008
France	Lac d'Annecy	45.86	6.17	1993-2008
France	Lac de Divonne les Bains	46.36	6.14	1993-2008
France	Lac de Machilly	46.26	6.33	1993-2008
France	Lac du Bourget	45.73	5.87	1993-2008
France	Léman français	46.44	6.56	1993-2008
France	L'Etang de Bresse	46.31	5.11	1993-2008
France	Miribel Jonage Grand Large	45.82	4.95	1993-2008
France	Pierre Bénite	45.70	4.82	1993-2008
France	Printe-Garde	44.80	4.78	1993-2008
France	Rhône, Seyssel	45.96	5.83	1993-2008
France	Rhône, St Romain en Gal	45.54	4.86	1993-2008
France	Rhône: l'Etournel	46.13	5.95	1993-2008
France	Roche de Glun	45.01	4.84	1993-2008
France	Saône	46.08	4.81	1993-2008
France	St Nazaire en Royans	45.06	5.25	1993-2008
France	Vallée de l'Ain et plans d'eau annexes	46.05	5.34	1993-2008
France	Villerest	45.99	4.04	1993-2008
Switzerland	Aare: Biel-Olten	47.21	7.53	2002-2007
Switzerland	Aare: Wohlensee	46.97	7.33	2002-2007
Switzerland	Bielersee	47.08	7.17	2002-2007
Switzerland	Bodensee-Obersee	47.65	9.18	2002-2007
Switzerland	Bodensee-Untersee	47.47	9.47	2002-2007
Switzerland	Hallwilersee	47.28	8.22	2002-2007
Switzerland	Hochrhein: Aaermündung-Basel	47.56	7.58	2002-2007
Switzerland	Hochrhein: Rheinklingen-Aaremündung	47.67	8.81	2002-2007
Switzerland	Lac de Morat	46.91	7.05	2002-2007
Switzerland	Lac de Neuchâtel	46.91	6.87	2002-2007
Switzerland	Lac Léman	46.44	6.56	2002-2007
Switzerland	Limmat	47.44	8.39	2002-2007
Switzerland	Reuss	47.27	8.32	2002-2007
Switzerland	Rhône: an aval de Genève	46.20	6.14	2002-2007
Switzerland	Thunersee	46.68	7.72	2002-2007

Switzerland	Vierwaldsättersee	46.99	8.34	2002-2007
Switzerland	Zugersee	47.13	8.49	2002-2007
Switzerland	Zurichsee	47.22	8.75	2002-2007
